# Informal payments in health facilities in Peru in 2018: Analysis of a cross-sectional survey

**DOI:** 10.1371/journal.pgph.0001837

**Published:** 2024-01-19

**Authors:** Laura Espinoza-Pajuelo, Patricia Mallma, Hannah Hogan Leslie, Patricia Jannet García

**Affiliations:** 1 Faculty of Public Health and Administration, Epidemiology Department, Universidad Peruana Cayetano Heredia, Lima, Peru; 2 Division of Prevention Science, Department of Medicine, University of California, San Francisco, San Francisco, CA, United States of America; Indian Institute of Technology Bombay, INDIA

## Abstract

**Background:**

The Latin American region demonstrates the lowest levels of trust in health systems globally. Institutional corruption is a major factor in eroding trust. Corruption in health services, including extracting bribes and informal payments from patients, directly harms health outcomes and weakens services intended as public goods. In this study, we aim to characterize the frequency and distribution of informal payments within public health services in Peru.

**Methods:**

We conducted a secondary analysis of a nationally representative cross-sectional survey, the 2018 National Household Survey of Living Conditions and Poverty, and identified all individuals reporting health insurance from the Ministry of Health (SIS-MINSA) or Social Security (ESSALUD). We defined self-reported informal payments in 2 ways: 1) being asked to pay a bribe at a health establishment in the past year (direct method), and 2) creating an overall indicator for non-zero cost of care for services that should be free (indirect method). We used descriptive statistics to quantify informal payments and bivariate analysis to identify sociodemographic characteristics of those most frequently reporting such payments.

**Findings:**

132,355 people were surveyed, including 69,839 (52.8%) with coverage from SIS-MINSA and 30,461 (23.03%) from ESSALUD. Less than 1% of participants directly reported informal payments, either at SIS-MINSA services (0.22%); or at ESSALUD (0.42%). Indirect reporting was more prevalent, including up to 10% of surgery patients and 17% of those hospitalized in SIS-MINSA facilities. Wealthier patients (19%) were more likely to report such payments.

**Interpretation:**

While direct reporting of bribery was uncommon, we found moderate prevalence of informal payments in public health services in Peru using an indirect assessment method. Indirect reporting may exceed direct reporting due to difficulty in distinguishing appropriate and inappropriate payments, fear of reporting health care workers’ behavior, or social tolerance of informal payments. Informal payments were more common among those with greater financial capital, indicating they may obtain enhanced services. Further research on patients’ perception and reporting of informal payments is a key step towards accurate measurement and evidence-based intervention.

## Introduction

Corruption affects health systems around the world [1–5]. According to García [6], the health sector is an attractive sector for corruption due to its complexity and the asymmetry of power between users and providers. Corruption in service delivery can be classified in six common types: absenteeism, informal payments, embezzlement, corruption during service provision (actions or medical treatments that were not necessary and that generate an economic or particular benefit to a third party), favoritism, and manipulation of data. Many forms of corruption, such as the diversion of funds, bribes as economic barriers to care, health personnel who do not attend at the established hours and do not provide the care that the population should receive, make it much more difficult to implement universal health coverage (UHC) [7]. Corruption in health systems limits access to health services and weakens all the dimensions of UHC: limiting access, undermining quality, increasing costs, and worsening inequity, each of which impedes health systems from delivering on the promise of better health for all [8]. Corruption in the health sector is not only a legal or economic phenomenon, but a social phenomenon: a set of practices that are embedded and normalized in the routine experience of health service users [9, 10]. Poor oversight systems and weak accountability mechanisms are associated with greater corruption in health systems [9].

In Peru, as in many Latin American countries, oversight systems and accountability are complicated by the fragmented and inequitable nature of the health system [11]. In 1975, Peru initiated a modern organization of its health system, becoming the first country in South America to do so [12]. However, the lack of a state policy led to inefficient performance, discontinuity, and a lack of assessment. For this reason, it was not until 1997–1998 that efforts to equalize health care access were implemented through national maternal and child health care programs. A public health insurance program for vulnerable populations and those in poverty, SIS-MINSA, was implemented in 2002, expanded in 2009–2010 within a UHC initiative, and further expanded by a 2019 decree affiliating all uninsured residents within SIS-MINSA [13]. The health system nonetheless remains fragmented, with 70% of the population covered by SIS-MINSA, 20% covered through the Social Security System (ESSALUD) through the Ministry of Labor, and small fractions covered through the armed forces and police and the private sector [14]. Health care services are provided separately within each of these sectors. This fragmentation informs the factors that foster corruption, such as weak regulatory oversight in healthcare facilities and personnel, complex bureaucratic procedures and excessive paperwork, lack of transparency in procurement processes for medical supplies and equipment and, one of the most harmful factors, the existence of a culture of impunity, in which the corrupt are not held accountable [15].

A precursor to accountability is accurate measurement [8]. However, measurement of forms of corruption such as informal payments is not yet standardized [16]. While multiple definitions of such payments have been proposed, a systematic review [17] recommends, “a direct contribution, which is made in addition to any contribution determined by the terms of entitlement, in cash or in-kind, by patients or others acting on their behalf, to health care providers for services that the patients are entitled to” [2] as the most clear and neutral definition. Operationalizing services to which patients are entitled in a specific health system is essential to capturing informal payments.

Informal payments in healthcare can occur at various points during user’s care process. This could involve offering under-the-table payments to healthcare providers in exchange for faster or more favorable treatment. Also, patients could pay to shorten waiting time or bypass queues at services, to obtain medicines or medical supplies in short supply, or to accelerate access to surgical services. In some cases, those payments take the form of gifts or non-monetary goods, which may come from people who are not necessarily sick, but who need other types of services such as falsification of medical documents [18–20]. Informal payments have important repercussions on the accessibility of health services, especially for the population living in vulnerable situations. A prior study in 33 low-income countries in Africa found informal payments provided an advantage to those with more resources and hence exacerbated disparities in access to health care between poor and rich as well as disparities between regions [21].

Understanding the prevalence of informal payments, their distribution across health services, and the factors associated with making such payments is an important step in ensuring accountability within health systems and improving the quality and equity of health care services. Measurement approaches to understand informal payments include both qualitative and quantitative methods, particularly household survey records and corruption surveys. For instance, in Peru, a 2018 study estimated the prevalence of informal payments in users of the SIS-MINSA public health insurance from 2008 to 2010 using information collected in the National Household Survey of Living Conditions and Poverty (ENAHO). This study found a prevalence of expenditures outside the tariff framework and during the health care process in all 25 regions of the country. It was also shown that the frequency of SIS-MINSA users who made these payments at some point during their process of care increased from 27% in 2008 to almost 35% in 2010 [22]. Since this time, ENAHO has added modules particularly focused on the evaluation of the population’s experience with government/public entities to assess governance directly asking for bribes or illegal situations.

In this study, we aim to describe prevalence of informal payments in public health services in Peru following the expansion of public health coverage, to identify the types of health services where informal payments are most prevalent, and to characterize individual health care users most susceptible to informal payments.

## Methods

### Study setting

Peru’s health system has two sectors, public and private. The public sector is divided into the subsidized regime (*Seguro Integral de Salud*, SIS-MINSA) and the direct contributive regime or social security (ESSALUD).

The Universal Health Coverage Law enacted in 2010 to guarantee the full right to health states that the entire population in the territory must have health insurance that covers access to the list of insurable conditions, which includes preventive, promotional, recuperative and rehabilitation services.

### Data source

We conducted a secondary analysis of the Opinion Module and the Health Module of the ENAHO 2018 cross-sectional survey. ENAHO 2018 was conducted by National Institute of Statistics and Informatics of Peru (*INEI* in Spanish; https://m.inei.gob.pe/); data are available through the *Datos Abiertos* (Open Data) platform of the Peruvian government.

This survey considers as a target population all private dwellings and household members, divided into three groups according to the level of relationship: head of household (over 18 years old), spouse (over 12 years old) and other members, residing in rural and urban areas at the national level. For the purposes of the study, we limited the target population of the ENAHO to those affiliated with the Comprehensive Health Insurance (SIS-MINSA) or Social Security (ESSALUD). The annual size of the ENAHO 2018 sample is 39 820 individual households, corresponding to 24 308 households in urban areas and 15 512 households in rural areas. The sample of clusters at the national level is 5 752, corresponding to 3 813 clusters in urban areas and 1 939 clusters in rural areas.

### Measures

There is no single gold standard measure for informal payments [17]. We used two complementary methods of assessment included in the ENAHO questionnaire to measure informal payments in Peru. The first was the direct method, which consisted of reporting being asked to pay a bribe at a public health establishment in the past year. This question was asked of participants 18 years of age and over who received care in any SIS-MINSA or ESSALUD facility in the last 12 months. Respondents were asked if they had been asked for a bribe or gift and separately if they had given one. We considered answering yes to either question as an indicator of informal payments, and we classified within this outcome whether payments had been solicited and/or provided.

The second one was the indirect method; we calculated reporting any type of informal payment or bribe among all affiliates of SIS-MINSA and ESSALUD who used specific health services within the past 12 months. Services included on the questionnaire were: medicines in the last 4 weeks; glasses and contraception in the last 3 months; inpatient attention and surgical intervention in the last year. Based on national policy in 2018, these services should be provided without charge to SIS-MINSA and ESSALUD affiliates. Since recall periods were shorter for services like medication use, this indicator may underestimate informal payments on an annual basis.

The independent variables were sociodemographic characteristics of the population: sex, age, degree of education, poverty, geographical region, and marital status. Educational attainment is defined by the Peruvian Ministry of Education: primary and secondary education constitute elementary, higher education in technological institutes or universities is considered undergraduate level, and masters and doctorate studies are postgraduate. Those who did not complete any of the mentioned levels were considered as uneducated. Poverty is defined by INEI, the official statistical agency in Peru that is responsible for collecting, analyzing, and disseminating data on various aspects of the country, including poverty. The 2018 classification defined poverty as monthly per capita expenditure less than the cost of a basic food and non-food consumption basket (approx. 95 USD).

### Statistical analysis

We utilized descriptive statistics to expound on the sample of users of healthcare services in the government facilities and to provide a report on the prevalence of informal payments. Additionally, we conducted a bivariate analysis using Fisher’s exact test (for categorical variables) and linear regression (for continuous characteristics) to compare frequency of informal payments across sociodemographic characteristics for each type of health service. Our level of significance was set at a p-value less than 0.05. The objective of our study was to characterize the groups that are most affected by informal payments, and we applied descriptive bivariate analyses accordingly [23].

ENAHO uses a stratified, multi-stage probability sampling approach. It divides the country into geographic strata and selects clusters in the first stage, followed by systematic sampling of households within selected clusters. Then, trained interviewers visit selected households to collect data through structured questionnaires. In cases of missing data, imputation techniques like the Hot Deck method are used to estimate missing values. ENAHO calculated survey weights based on the sample design considering probabilities of selection and nonresponse; weights are scaled to population size by age and sex group. We accounted for stratified sampling and sample weights in all analyses using the *svy* setting in Stata.

We used the statistical program STATA version 17.0 for the analysis.

### Ethics statement

This research project was approved by the Institutional Ethics Committee of the Universidad Peruana Cayetano Heredia under code SIDISI 201297 in January 2020. Verbal consent was obtained as part of the original study from ENAHO 2018. Ethical review of this secondary analysis of deidentified data confirmed no further consent was required.

We followed STROBE guidelines for reporting of cross-sectional studies (S1 Appendix).

## Results

Of the 39,820 households sampled, ENAHO surveyed 132,355 people. Within them, 69,839 (52.80%) were affiliated to SIS-MINSA and 30,461 (23.03%) to ESSALUD (Table 1). The population covered by SIS-MINSA was poorer and more rural than ESSALUD affiliates (32.56% vs 5.71% in poverty, p<0.001, and 36.80% rural vs. 4.65%, p<0.001). In addition, ESSALUD affiliates were more likely to have completed undergraduate education (36.23% ESSALUD vs. 9.53% MINSA p<0.001).

**Table 1:** Demographics characteristics of ESSALUD and SIS-MINSA user’s.

	ESSALUD N = 30,461 (%)	SIS-MINSA N = 69,839 (%)	Total N = 132,355 (%)	Chi square p value
**Sex**				
Female	50.91	54.64	51.49	<0.001
Male	49.09	45.36	48.51	
**Age (mean; SD)**	38.13 (23.46)	31.03 (23.03)	33.71 (22.49)	< .001
**Poverty level**				
Poor	5.71	32.56	20.42	<0.001
Non poor	94.29	67.44	79.58	
**Area**				
Urban	95.35	63.20	78.10	<0.001
Rural	4.65	36.80	21.90	
**Level of instruction:**	**N = 29,422**	**N = 65,923**	**N = 126,711**	
None	3.12	9.72	6.37	<0.001
Elementary	56.93	80.71	70.60	
Undergraduate	36.23	9.53	21.84	
Graduate	3.72	0.04	1.19	
**Marital status**	**N = 25,051**	**N = 52,001**	**N = 105,175**	<0.001
Single	29.37	34.66	36.03	
Non-single	70.63	65.34	63.97	

**Fig 1:**
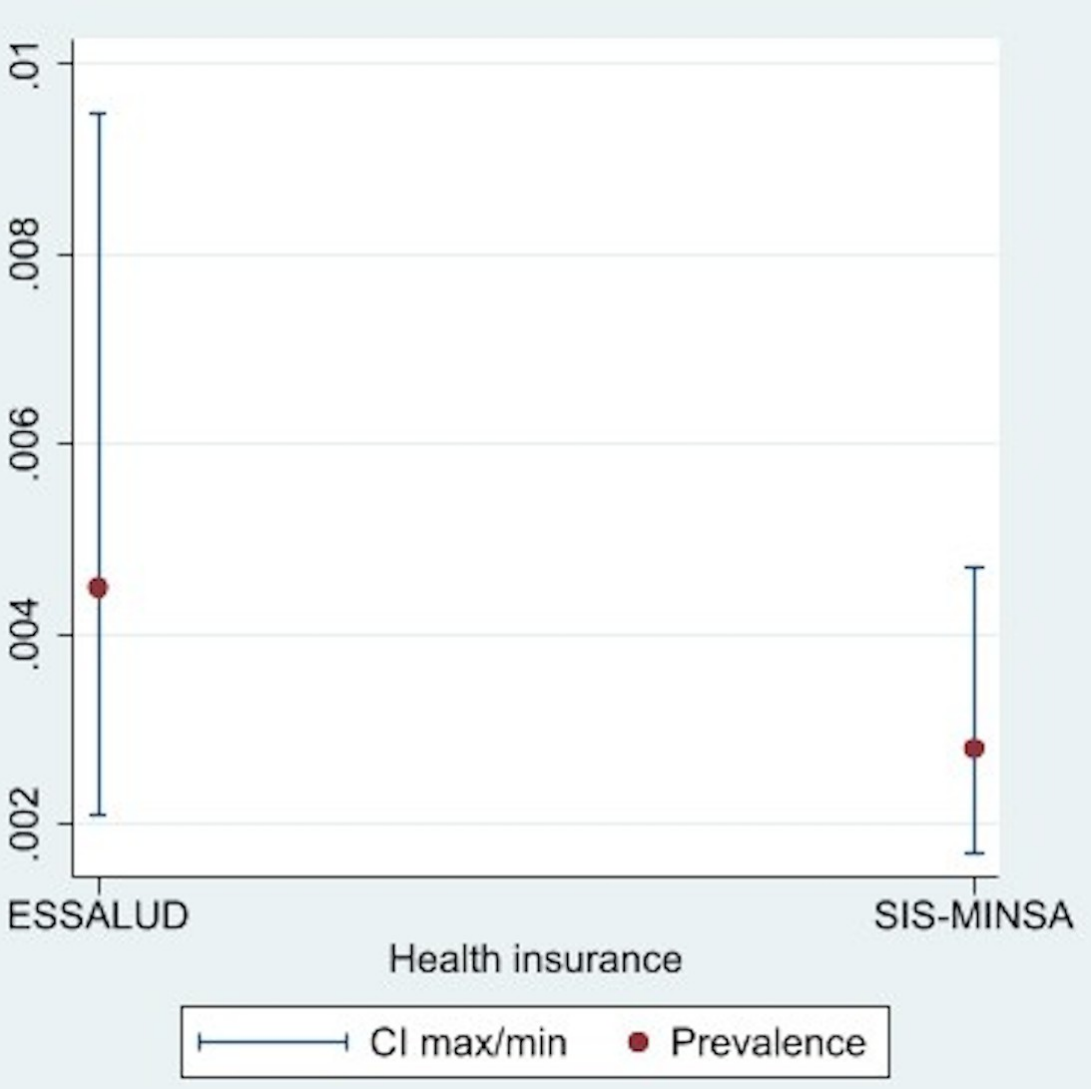
Prevalence of informal payments in ESSALUD and SIS-MINSA ‐ direct method approach.

To evaluate the prevalence of informal payments according to the direct approach, we considered the response of the heads of household who also had public health insurance, so we analyzed the data of 3,964 insured in ESSALUD and 11,458 in SIS-MINSA. The prevalence of informal payments was 0.42% within ESSALUD and 0.22% within SIS-MINSA (Fig 1) using the direct approach.

Over 99% of respondents from both ESSALUD and MINSA reported neither being asked nor providing informal payments. Among ESSALUD affiliates, 0.56% reported being asked for an informal payment (of whom 41% provided one) and 0.19% of patients reported making informal payments even when not asked to do so by staff or providers. A similar trend was observed among SIS-MINSA affiliates, with 0.64% asked for an informal payment (and 22% providing one) and only 0.08% of SIS-MINSA patients making informal payments without being prompted to do so during their care.

**Fig 2:**
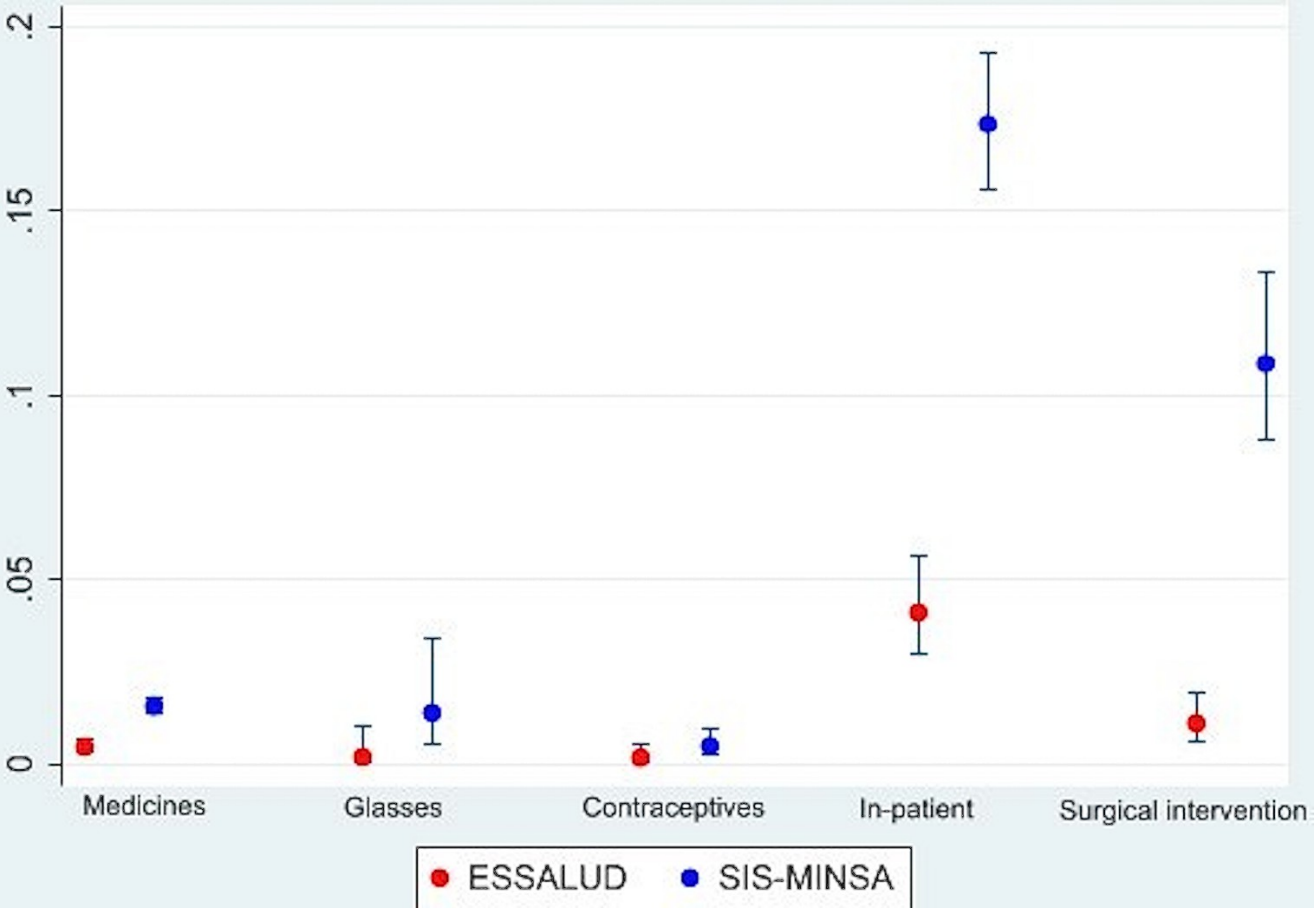
Informal payments prevalence in ESSALUD and SIS-MINSA health services ‐ indirect method approach.

Using the indirect method of reporting cost for a service that should be no-cost, (Fig 2) within SIS-MINSA services, the highest prevalence of informal payments made for users in the last year occurred during in-patient services (17.37% of SIS-MINSA users, 4.11% of ESSALUD users) and surgical intervention (10.86% within SIS-MINSA, 1.11% within ESSALUD).

We characterized the population that most affected in services with prevalence of informal payments > 4%. We found that within the SIS-MINSA hospitalization services, those who are not in poverty and who live in urbanized areas were the ones who had made informal payments during their care process (Table 2). We did not identify statistically significant differences in characteristics of ESSALUD patients reporting and not reporting informal payments.

**Table 2:** Sociodemographic characteristics of population in high-reported informal payments health services.

	In-patient						Surgical intervention		
	ESSALUD N = 1,793 (%)		Fisher exact test p value	MINSA N = 3,042 (%)		Fisher exact test p value	MINSA N = 1,539 (%)		Fisher exact test p value
	Yes	No		Yes	No		Yes	No	
**Sex**			0.709			0.136			0.290
Female	3.83	96.17		17.16	82.84		10.66	89.34	
Male	4.17	95.83		19.42	80.58		8.93	91.07	
**Level of instruction**			0.944			0.086			0.056
None	3.77	96.23		14.34	85.66		10.74	89.26	
Basic	3.76	96.24		18.44	81.56		8.54	91.46	
Undergrad	4.29	95.71		18.60	81.40		14.29	85.71	
Graduate	3.90	96.10		100	0.00		0.00	100	
**Poverty level**			0.720			0.001*			0.196
Poor	1.96	98.04		13.33	86.67		7.17	92.83	
Non-poor	4.02	95.98		19.07	80.93		9.87	90.13	
**Stratum**			0.845			0.016*			0.050
Urban area	3.94	96.06		19.27	80.73		10.63	89.37	
Rural area	4.17	95.83		15.88	84.12		7.63	92.37	
**Marital status**			0.588			0.330			0.132
Single	4.60	95.40		19.72	80.28		12.21	87.79	
Non-single	3.84	96.04		17.79	82.21		9.05	90.95	
			**Linear regression p value**			**Linear regression p value**			**Linear regression p value**
**Age (mean; SD)**			0.780			0.868			0.205
	42.63 (24.67)	43.78 (24.09)		35.43 (21.22)	35.20 (22.73)		35.99 (16.87)	38.70 (20.54)	

## Discussion

The present study analyzed the prevalence of informal payments within those covered by public health services in Peru through a direct method and an indirect method. We found that, when asked directly, the prevalence of informal payments was very low, less than half a percent in each sector. However, using the indirect method, the reporting percentages were much higher, especially in the in-patient service (MINSA: 17.37%, ESSALUD: 4.11%) and surgical intervention (MINSA: 10.86%; ESSALUD: 1.11%). Our analysis of individuals making informal payments in these services revealed a statistically significant relationship between such payments and two characteristics: urban residence and a classification as non-poor based on their living conditions.

Our results suggest a decline in prevalence compared to the analysis of the 2010 national study [22]. It is possible these results suggest a true decline in informal payments over time, given the expansion of social security and the broader availability of affordable services in the public health system. However, the difference may also be related to changes in measurement: although both analyze informal payments with almost the same set of questions from the cross-sectional national survey ENAHO, the survey in 2010 and 2018 version has changed such that 2010 items focus on the service/product received in the previous 4 weeks and include details on the type of facility and professional interacting with the patient. The 2018 survey used a range of recall periods across health services and did not include details on the health personnel or the type of facility.

The extremely low prevalence of informal payments reported directly is a striking finding. Within the same nationally representative survey, individuals who were directly asked about informal payments reported a much lower frequency compared to those who only indicated that they had made payments while insured. This could reflect social desirability, in a context such as Peru, where corruption is widespread and social norms may conflict when asking about corrupt behavior or complicity in corrupt behavior [24]. In this sense, it’s suggested to generate data by selecting other strategies that reduce the discomfort of respondents when having to answer a sensitive question. It could be achieved using alternatives approaches of recollection of informal situations like the indirect method for informal payments, which take into consideration participants who attended public services and requested services which are free and fully covered by law but are willing to pay to obtain free access to full healthcare coverage and medications. Items that do not directly mention “informal”, “illegal” or “corruption” reduce the potential risk-perception to the respondents. A challenge in operationalizing informal payments is clarity on the benefits to which the patient is entitled free of charge [17]. Differentiating between informal payments and legitimate out-of-pocket payments may be difficult due to a lack of patients’ awareness of their legal rights and coverage entitlements. In that sense, it may be valid to assume that all transactions where patients report being not given any information about official co-payments rates or fees are informal [4, 25, 26]. Future efforts to measure informal payments may consider assessment of respondents’ awareness of entitlements and expected payments.

Services with higher prevalence of informal payments included in-patient care from both SIS-MINSA and ESSALUD, and surgical intervention within SIS-MINSA. Multiple factors may account for this finding. Users may be more motivated to pay for higher quality care for high acuity services than for primary care, particularly when such services are in short supply. A significant percentage of the Peruvian population lacks proper access to the health center of high level of complexity, leading to barriers in effective care delivery [27]. Inpatient services often involve more complex and specialized care and more specific resources and medications; they are less familiar to patients than routine primary care. These factors may lead to higher expectations from patients and a greater willingness to make informal payments [28, 29]. Although there is no evidence patients in Peru must buy their own supplies, this has been demonstrated in other countries which also face structural health systems issues. In those countries, patients or their relatives are required to contribute essential materials as medicines, medical supplies, injections, vaccines, diapers, and even non-medical supplies for their surgical intervention or their in-patient stay [30]. These factors create an environment where patients feel compelled to make informal payments to receive the care they need.

Within SIS-MINSA, less poor individuals and those in urban areas were more likely to make informal payments. Although more than half of the population is affiliated to SIS-MINSA, which is the insurance that covers those who are in a situation of vulnerability and who do not have other health insurance, this does not mean that affiliates are without sources of income with which they could pay for health care. According to the World Labor Organization, 75% of the workers in Peru are informal [31], so they would not have a social insurance granted by their employer, thus belonging to the SIS-MINSA. This means that within this insurance there are people who have economic resources, and although these resources may be limited, they could agree to make informal payments because these payments would be significantly lower compared to expenses in a private facility [26, 32]. Another possibility is that health care providers do not approach patients with limited resources for informal payments. Finally, some researchers have hypothesized that informal payments could lead to "redistribution" among users, with welfare/administrative staff playing a "Robin Hood" role, subsidizing the poor and charging the rich [21]. One of the assumptions for this to occur is that health care personnel represent a "collection agency for medical charities" that solicits the better off above marginal cost and uses the proceeds to provide care for the poorest. However, no studies to date have substantiated the idea that the poorest actually receive a benefit in access, quality, or satisfaction.

### Study strength and limitations

Since this is a secondary analysis, we used the questions that had already been asked, and adapted the variables to define informal payments. Details on the informal payments, such as amount, recipient(s), and presence of coercion, are not available [28]. In addition, we have evaluated one side of the problem; however, it would be ideal to know the attitude of health personnel in these cases, as well as the motivations they could have for requesting informal payments.

The main strength of our study is the update of the analysis of the prevalence of informal payments collected annually in SIS-MINSA and ESSALUD establishments since 2010. The present study implemented two approaches for measuring informal payments to provide estimates of informal payments at the national level. Furthermore, it allows us to inform the ways that the government measures sensitive topic such as corruption. Patients may not be aware of the benefits to which they are entitled; increasing communication around covered services and assessing individuals’ awareness of such coverage is a strategy to consider. More accurate measurements and further research is necessary to report the real estimate of informal payments. This study’s results are of interest and useful to share with government health entities towards the generation of evidence-based actions for the benefit of the population.

## Supporting information

S1 Appendix STROBE statement—checklist of items that should be included in reports of *cross-sectional studies*. (DOC)
